# Comparative Analysis of MBNL1 Antibodies: Characterization of Recognition Sites and Detection of RNA Foci Colocalization

**DOI:** 10.3390/genes16060658

**Published:** 2025-05-29

**Authors:** Yoshitaka Aoki, Ai Ohki, Motoaki Yanaizu, Yoshihiro Kino

**Affiliations:** Department of RNA Pathobiology and Therapeutics, Meiji Pharmaceutical University, 2-522-1, Noshio, Kiyose-shi 204-8588, Tokyo, Japanm-yana@my-pharm.ac.jp (M.Y.)

**Keywords:** MBNL1, antibody, RNA foci, myotonic dystrophy

## Abstract

**Background/Objectives:** MBNL1 is an RNA-binding protein involved in RNA metabolism, including splicing. It colocalizes with RNA foci, a pathological hallmark of myotonic dystrophy, and plays a central role in its disease mechanism. Moreover, MBNL1 has been implicated in other neuromuscular disorders and cancers. In these pathological and biochemical studies, the detection of MBNL1 using antibodies is essential. Given that MBNL1 has multiple splicing-derived isoforms, different antibodies may recognize distinct isoforms. This study aims to compare six commercially available antibodies regarding their specificity in Western blotting, colocalization with RNA foci, and suitability for immunoprecipitation. **Methods**: Western blot analysis was performed using MBNL1 isoforms and deletion mutants expressed in HEK293 cells, as well as endogenous MBNL1 from various cell lines. RNA fluorescence in situ hybridization (FISH) and immunofluorescence (IF) were conducted in DM1 model cells and patient-derived fibroblasts to assess MBNL1 colocalization with RNA foci. Immunoprecipitation experiments were performed in HEK293 cells to evaluate antibody suitability for protein isolation. **Results**: Western blot analysis revealed that different antibodies target distinct regions of MBNL1, with three recognizing exon 3 and the remaining antibodies recognizing exon 4, exon 5, and exon 6, respectively. In the FISH-IF experiments, the clarity of RNA foci colocalization varied depending on the antibody used, with some antibodies failing to detect colocalization. The immunoprecipitation analysis showed that four antibodies were able to isolate endogenous MBNL1. **Conclusions**: This study clarifies the recognition properties and application suitability of MBNL1 antibodies, providing a valuable resource for research on MBNL1-related diseases and RNA metabolism.

## 1. Introduction

Muscleblind-like 1 (MBNL1) is an RNA-binding protein that regulates various aspects of RNA metabolism, including alternative splicing, microRNA biogenesis, RNA transport, and RNA stability [1,2,3,4,5]. It is an ortholog of muscleblind, which is involved in eye and muscle development in *Drosophila melanogaster* [6,7] and has two paralogs in humans: MBNL2 and MBNL3 [8]. While MBNL1 and MBNL2 are broadly expressed across various tissues, MBNL1 is predominantly expressed in skeletal and cardiac muscles, whereas MBNL2 is highly expressed in the brain. MBNL3 is mainly expressed in the placenta, lever, and fetal tissues. MBNL1 has been implicated in several neuromuscular disorders, with myotonic dystrophy (DM) being the most extensively studied conditions.

Myotonic dystrophy type 1 (DM1) is an autosomal dominant neuromuscular disorder caused by the abnormal expansion of CTG repeat sequences in the 3′ untranslated region (3′UTR) of the *DMPK* gene [9]. The normal repeat number ranges from approximately 5 to 37, whereas in DM1 patients, the expansion can reach from 50 to several thousand repeats. Larger repeat numbers are associated with earlier disease onset and greater severity of symptoms. When the repeat number exceeds 1000, congenital DM1 can develop. The primary symptoms of DM1 include myotonia, muscle atrophy, cataracts, cardiac conduction defects, and insulin resistance. Myotonic dystrophy type 2 (DM2), which presents symptoms similar to those of DM1, is caused by an abnormal expansion of CCTG repeat sequences within intron 1 of the *CNBP* gene [10]. Unlike DM1, DM2 does not show a clear correlation between repeat length and disease severity, nor does it present a congenital form. Both DM1 and DM2 are considered RNA gain-of-function diseases. The abnormal expansion of CTG or CCTG repeats leads to the production of CUG- or CCUG-repeat RNA, which forms nuclear RNA foci. MBNL proteins excessively accumulate in these RNA foci, leading to functional inhibition [8,11]. Loss of MBNL1 function causes splicing abnormalities in multiple genes, including the insulin receptor (*IR*), chloride channel (*CLCN1*), and sodium channel (*SCN5A*) [12,13,14]. In *Mbnl1* knockout mice, DM1-like splicing abnormalities, myotonia, pathological muscle defects, and cataracts have been reported [15], suggesting a direct role in DM1 pathogenesis. Meanwhile, *Mbnl2* knockout mice exhibit cognitive impairments and sleep disturbances [16]. MBNL proteins redundantly regulate common target genes, and simultaneous depletion of MBNL1 and MBNL2 exerts a stronger effect than individual deletions [17].

In addition to DM1 and DM2, abnormal localization of MBNL1 has been reported in several other neuromuscular diseases. CTG repeat expansions have been identified in both Huntington’s disease-like 2 and Fuchs endothelial corneal dystrophy, similar to DM1. In these disorders, MBNL1 colocalizes with RNA foci [18,19]. Similarly, fragile X-associated tremor/ataxia syndrome and various types of oculopharyngodistal myopathy are characterized by GGC repeat expansions, where MBNL1 colocalizes with ubiquitin-positive nuclear inclusions [20,21,22,23]. MBNL1 is also involved in various cancers (for a review, see [24]). However, the extent to which MBNL1 contributes to the pathogenesis of these diseases remains unclear compared to DM.

MBNL1 contains four C3H-type zinc finger motifs and specifically binds to YGCY sequences (Y = C or U) [2,25]. The CUG and CCUG repeat sequences resemble this recognition motif and contain multiple MBNL1-binding sites. MBNL1 exists in multiple splicing variants. MBNL1 lacking exon 1 loses its zinc finger motifs, resulting in reduced splicing regulatory activity and protein instability [26]. Of the four zinc finger motifs, the first two and the last two function as pairs. The majority of the linker region connecting the first two and the last two is encoded by exon 3, which undergoes alternative splicing. Skipping of exon 3 leads to a reduction in RNA-binding capacity and splicing regulatory function [27,28]. Additionally, the C-terminal region contains multiple alternatively spliced exons, with the inclusion of exon 5 contributing to nuclear localization signals [27,28]. In contrast, exon 7 inclusion promotes self-association [27]. Thus, the function and properties of MBNL1 are significantly influenced by alternative splicing. Furthermore, exons such as exon 1 and exon 5 are regulated by MBNL1 itself or its paralogs [26,28,29].

As mentioned above, MBNL1 has been implicated in numerous diseases, and its pathological and biochemical alterations are actively being studied. In these studies, the detection of the MBNL1 protein using antibodies is essential. It is important to understand the characteristics of the antibodies used, as the detection of MBNL1 may vary depending on the specific antibody employed. In addition to possessing multiple isoforms, MBNL1 also has paralogs with overlapping functions [30]. Therefore, it is essential to determine which MBNL1 isoform is detected and whether any cross-reactivity occurs with other paralogs. Although several commercial MBNL1 antibodies are available, it has not been thoroughly examined which regions of MBNL1 each antibody recognizes, which isoforms they detect, or whether they selectively recognize specific isoforms in particular cell types. In this study, we aimed to compare six commercially available MBNL1 antibodies (Table 1) and evaluate their specificity in Western blotting and RNA foci detection.

## 2. Materials and Methods

### 2.1. Plasmid Construction

The human cDNA sequences of MBNL1, MBNL2, and MBNL3 have been described previously [28]. To generate a novel MBNL1 deletion mutant (1–138 and 1–161), polymerase chain reaction (PCR) amplification was performed using a forward primer containing a BamHI sequence and a reverse primer with an XhoI sequence. The amplified product was digested with BamHI and XhoI then inserted into the BglII-SalI site of the pEGFP-C1 vector (Clontech, Mountain View, CA, USA).

### 2.2. Cell Culture

HEK293 (RCB1637, Riken BRC, Ibaraki, Japan), HeLa (#CCL-2, ATCC, Manassas, VA, USA), HeLa-CTG [31], Neuro2A (#CCL-131, ATCC), C2C12 (RCB0987, Riken BRC), and fibroblasts derived from a DM1 patient with a CTG1500 repeat (GM05151, Coriell Cell Repository, Camden, NJ, USA) were cultured in Dulbecco’s modified Eagle medium (DMEM, Invitrogen, Carlsbad, CA, USA) supplemented with 10% fetal bovine serum (FBS, Nichirei Biosciences, Tokyo, Japan) and 1% penicillin–streptomycin (Wako, Osaka, Japan) at 37 °C under 5% CO_2_. The establishment of HeLa-CTG cells has been previously reported. In addition, THP-1 cells (RCB1189, Riken BRC) were maintained in RPMI 1640 Medium with GlutaMAX supplement (Thermo Fisher Scientific, Waltham, MA, USA), supplemented with 10% FBS (Nichirei Biosciences) and 1% penicillin–streptomycin (Wako) at 37 °C under 5% CO_2_.

### 2.3. Transfection

For plasmid transfection, HEK293 cells were seeded in a 12-well plate one day before transfection. Opti-MEM (Gibco, Miami, FL, USA), Lipofectamine 2000 (Thermo Fisher Scientific), and 0.5 μg of plasmid DNA were added to each well. For siRNA experiments, siMBNL1-623 (gcacaaugauugacaccaaTT) was used for human cells, while siMbnl1-88 (ccagacacggaauguaaauTT) was used for mouse cells. The sequences represent the sense strand and consist of 19-mer RNA followed by dTdT. For siRNA transfection, cells were seeded in a 6-well plate one day before transfection, and Lipofectamine RNAiMAX (Thermo Fisher Scientific), Opti-MEM (Gibco), and 30 pmol of siRNA were added.

### 2.4. Sodium Dodecyl Sulfate-Polyacrylamide Gel Electrophoresis (SDS-PAGE)

The SDS-PAGE procedure was performed as described [31]. The antibodies used in this study are listed in Table 1. Full Western blot images are provided in Supplementary Figure S6.

### 2.5. Reverse Transcription-PCR (RT-PCR)

cDNA synthesized from each cell line was used as a template for PCR amplification of MBNL1/Mbnl1 using Blend-Taq (TOYOBO, Osaka, Japan) and the primers listed below. For human MBNL1, MBNL1-ex1-116Fw (5′-CTTCGAAAAGCTGCCAAGTTGAAAATGGACGAGTA-3′) and SalI-MBNL1-st-Rv (5′-AAAAAAGTCGACCTACATCTGGGTAACATACTTGTG-3′) were used. For mouse Mbnl1, Mbnl1-isoforms-Fw (5′-CCGGTGCCCCGTTGCAGCCCGT-3′) and Mbnl1-isoforms-Rv2 (5′-GATACCCATAATATCTGCC-3′) were used. PCR products were separated on an 8% polyacrylamide gel and stained with ethidium bromide (Wako). Gel images were captured using Luminograph III (ATTO, Tokyo, Japan). An uncropped image is provided in Supplementary Figure S6. For some major bands, amplified DNA was extracted from the gel following electrophoresis using a 2% agarose gel and subjected to Sanger sequencing by FASMAC.

### 2.6. Fluorescence In Situ Hybridization (FISH)

FISH was carried out as previously described [28,31]. HeLa-CTG cells were treated with doxycycline (Dox, Sigma, St. Louis, MO, USA) at a final concentration of 1.0 μg/mL in the culture medium. After 24 h, gene induction was confirmed by detecting red fluorescent protein (RFP) fluorescence using an EVOS M5000 microscope (Thermo Fisher Scientific). The probe Cy5-2OMe-(CAG)7 (Japan Bio Services, Saitama, Japan) was used. In addition to the aforementioned method using 4% paraformaldehyde fixation, acetone–methanol fixation was also employed. The procedure was conducted according to the previously reported method [32,33]; however, herring sperm DNA was not added to the hybridization solution.

### 2.7. Immunofluorescence Staining

Immunofluorescence staining was performed as previously described [34]. The antibodies used in this study are listed in Table 1. Immunofluorescence staining was conducted following FISH, and cells were permeabilized with PBS containing 0.1% Triton X-100 for 5 min. Colocalization of MBNL1 and RNA foci was analyzed using Fiji [35]. Based on FISH-IF images, several lines were first set within the nuclei as regions of interest. Fluorescent intensity line profiles were then obtained for both immunofluorescence and FISH images. Pearson correlation coefficients were calculated from these line profile values. For each antibody, at least 15 line profiles were obtained from three or more cell images. The resulting correlation coefficients were analyzed using the Kruskal–Wallis test, followed by Dunn’s test with Bonferroni correction. Statistical analysis was performed using Python (version 3.12.4).

### 2.8. Immunoprecipitation

HEK293 cells were cultured in 10 cm dishes, collected in radioimmunoprecipitation assay (RIPA) buffer containing 1× protease inhibitor, and sonicated for 2 min. The lysate was centrifuged at 16,100× *g* for 15 min to separate fractions. The supernatant was pre-cleared with Dynabeads Protein G (Thermo Fisher Scientific) and Dynabeads Protein A (Thermo Fisher Scientific) at 4 °C for 2 h. A portion of the pre-cleared lysate was collected as input, and the remaining lysate was incubated overnight at 4 °C with the following antibodies conjugated to their respective beads for immunoprecipitation: 3E7, 4A8, 66837-1-Ig, sc-58790, and mouse IgG were conjugated to Dynabeads Protein G, while ab45899, ARP41227, and rabbit IgG were conjugated to Dynabeads Protein A. After three washes in RIPA buffer containing protease inhibitors, the beads were mixed with SDS sample buffer and boiled. Immunoprecipitated MBNL1 was detected using ab45899 for Dynabeads Protein G and 4A8 for Dynabeads Protein A.

## 3. Results

### 3.1. Differential Detection of MBNL1 by Various Antibodies

**Figure 1:**
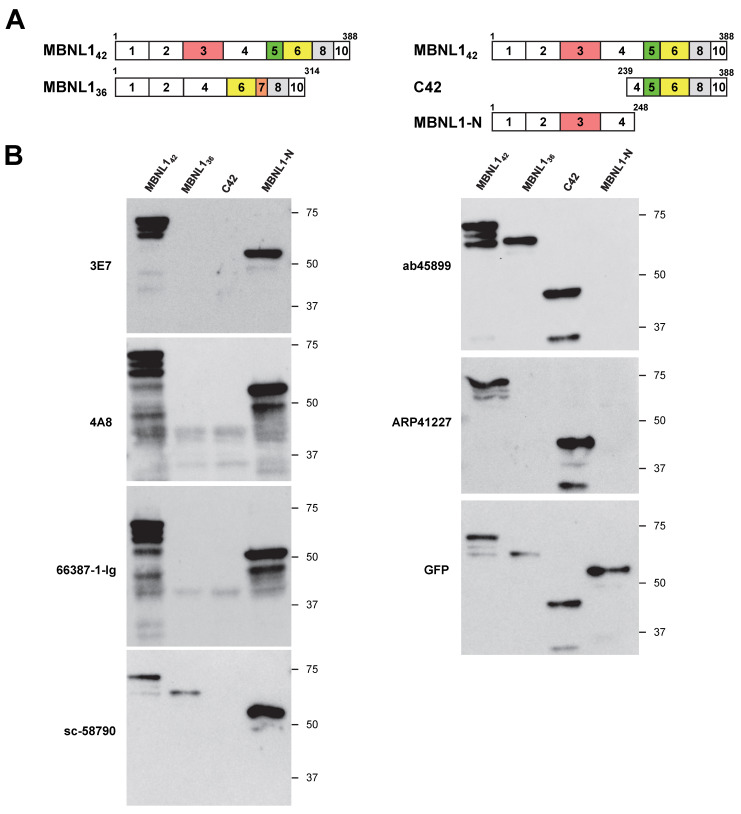
Western blot analyses utilizing various MBNL1 antibodies: (**A**) diagram depicting MBNL1 isoforms (MBNL1_42_ and MBNL1_36_) and deletion mutants (C42 and MBNL1-N), with squares representing exons; (**B**) Western blot analysis illustrating the results obtained using different MBNL1 antibodies.

In this study, we first aimed to determine whether six different antibodies recognize the N-terminal or C-terminal regions of MBNL1. Considering the possibility of isoform-specific recognition, we utilized four MBNL1 constructs with an N-terminal EGFP (enhanced green fluorescent protein) tag. The MBNL1 isoforms MBNL1_42_ and MBNL1_36_ contain exon 5 and exon 7, respectively (Figure 1A). C42 corresponds to the C-terminal region of MBNL1_42_, while MBNL1-N represents a deletion mutant containing the four zinc finger (ZnF) motifs within the N-terminal region of MBNL1, including exon 3. These constructs were transfected into HEK293 cells, and the reactivity of six antibodies was evaluated via Western blotting.

The antibodies 3E7, 4A8, 66837-1-Ig, and sc-58790 detected MBNL1-N but did not recognize C42 (Figure 1B), indicating that they all target the N-terminal region. Among these, 3E7, 4A8, and 66837-1-Ig did not detect MBNL1_36_, which lacks exon 3, suggesting that they specifically recognize the region encoded by exon 3. In contrast, sc-58790 detected MBNL1_36_, implying that it recognizes the N-terminal region but targets a site outside of exon 3. In contrast to these N-terminal-recognizing antibodies, ab45899 and ARP41227 detected C42 but not MBNL1-N (Figure 1B), demonstrating specificity for the C-terminal region. MBNL1_42_ and MBNL1_36_ possess distinct C-terminal regions due to alternative splicing. Interestingly, while ab45899 recognized both isoforms, ARP41227 did not detect MBNL1_36_, indicating that ARP41227 recognizes the exon 5 region, which is present in MBNL1_42_ but absent in MBNL1_36_. In summary, four antibodies (3E7, 4A8, 66837-1-Ig, and sc-58790) were found to recognize the N-terminal region, while two antibodies (ab45899 and ARP41227) were specific to the C-terminal region.

### 3.2. Mapping of MBNL1 Antibody Recognition Sites Using Deletion Mutants

**Figure 2:**
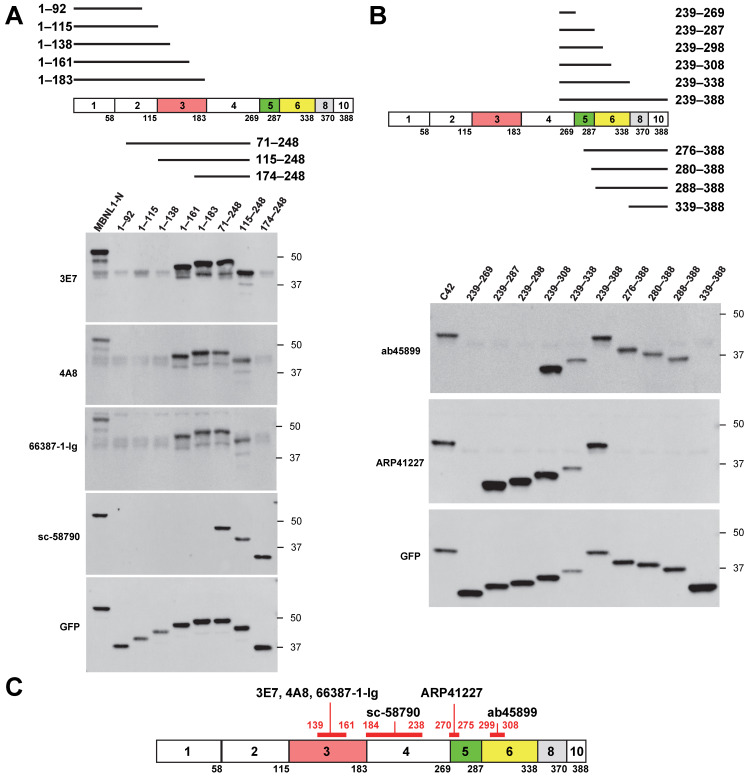
Identification of recognition sites for MBNL1 antibodies: (**A**) schematic representation of N-terminal deletion mutants alongside Western blot analysis of MBNL1 antibodies targeting the N-terminal region; (**B**) schematic representation of C-terminal deletion mutants and corresponding Western blot analysis of MBNL1 antibodies recognizing the C-terminal region; (**C**) diagram depicting the recognition sites of the tested MBNL1 antibodies.

Next, we used deletion mutants to precisely determine the recognition sites of various MBNL1 antibodies. Among the four antibodies that recognize the N-terminal region of MBNL1, 3E7, 4A8, and 66837-1-Ig did not detect deletion mutants 1–138 and 174–248 but did detect 1–161 and 115–248 (Figure 2A). These results suggest that these antibodies recognize amino acids within the 139–161 region. Since this region is encoded by exon 3, this finding is consistent with the fact that these antibodies did not recognize MBNL1_36_, which lacks exon 3. In contrast, sc-58790 did not detect 1–183 or C42 (239–388) but did detect 174–248 (Figure 1B and Figure 2A), suggesting that it likely recognizes amino acids within the 184–238 region, encoded by exon 4.

Next, we examined the antibodies ab45899 and ARP41227, which recognize the C-terminal region (Figure 2B). ab45899 did not detect deletion mutants 239–298 or 339–388 but detected 239–308 and 288–338, suggesting that the 299–308 region, located within exon 6, is essential for its recognition. ARP41227 did not detect 239–269 or 276–388 but did recognize 239–287, indicating that it requires the 270–275 region within exon 5 for recognition. To further validate the recognition site of ARP41227, an additional mutant (270–308) was analyzed (Figure S1). Since ARP41227 reacted with the 270–308 mutant but not with the 276–388 mutant, these findings support the conclusion that the 270–275 region is essential for recognition. This suggests that ARP41227 recognizes a portion of exon 5, which is involved in nuclear localization. Consistently, ARP41227 detected MBNL1_42_, which includes exon 5, but did not detect MBNL1_36_, which lacks exon 5 (Figure 1B). The recognition sites of the antibodies are summarized in Figure 2C.

### 3.3. Comparison of Antibody Reactivity to MBNL Paralogs

**Figure 3:**
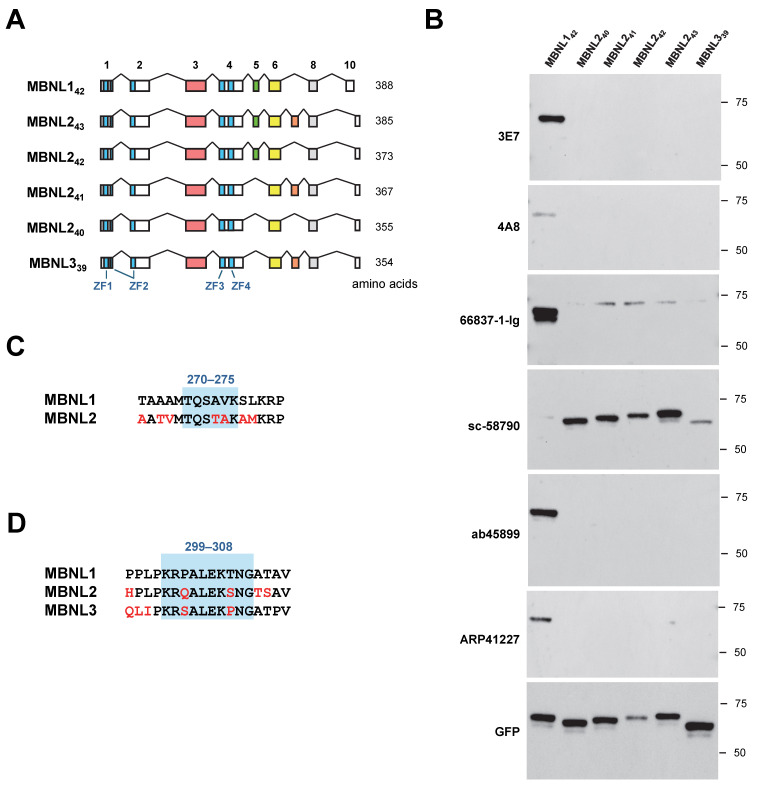
Cross-reactivity of MBNL1 antibodies with MBNL paralogs. (**A**) Schematic representation of MBNL1_42_, MBNL2 isoforms (MBNL2_40_, MBNL2_41_, MBNL2_42_, and MBNL2_43_), and MBNL3_39_. Blue boxes within the exons indicate the zinc finger motifs. (**B**) Western blot analysis of HEK293 cells transfected with MBNL2 and MBNL3 isoforms using MBNL1 antibodies. (**C**) Amino acid sequence of the putative recognition site of ARP41227 on exon 5. Corresponding sequence of MBNL2 is also shown. (**D**) Amino acid sequence of the putative recognition site of ab45899 on exon 6, together with corresponding sequences of MBNL2 and MBNL3.

HEK293 cells were transfected with EGFP-fused MBNL2 and MBNL3 (Figure 3A), and the reactivity of six antibodies used in this study was assessed via Western blotting. Among these antibodies, all except sc-58790 showed no cross-reactivity with MBNL2 or MBNL3 (Figure 3B). sc-58790 has been reported to recognize all three MBNL paralogs (MBNL1, MBNL2, and MBNL3); however, it detected MBNL2 most efficiently, while MBNL1 was only faintly detected.

The exon corresponding to MBNL1 exon 5 is present in MBNL2_42_ and MBNL2_43_, but ARP41227, which recognizes exon 5, did not detect these MBNL2 isoforms (Figure 3A,B). The region surrounding amino acids 270–275 in MBNL1 contains residues that are not conserved in MBNL2, which may contribute to differences in antibody reactivity (Figure 3C). Similarly, ab45899 exclusively detected MBNL1. The region of MBNL1 exon 6 recognized by this antibody contains amino acids that are not evolutionarily conserved in the corresponding sequences of MBNL2 and MBNL3 (Figure 3D). Notably, Pro301 and Thr306 are unique to MBNL1, suggesting that ab45899 may specifically recognize these residues.

### 3.4. Endogenous Expression Patterns of MBNL1

**Figure 4:**
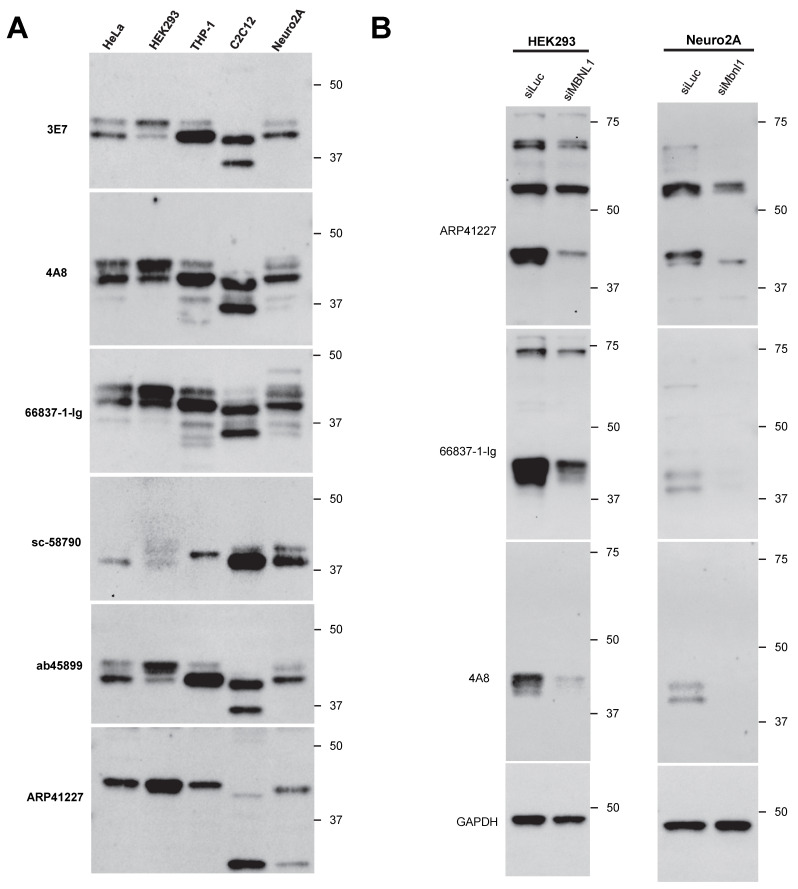
Detection of endogenous MBNL1 across different cell types: (**A**) detection of endogenous MBNL1 in HeLa, HEK293, THP-1, C2C12, and Neuro2A cells via Western blot analysis; (**B**) Western blot analysis of HEK293 and Neuro2A cells transfected with MBNL1-targeting siRNA.

Next, we examined the endogenous expression patterns of MBNL1 in several human- and mouse-derived cell lines using various antibodies. HeLa, HEK293, THP-1, C2C12, and Neuro2a cells were analyzed (Figure 4A), revealing differences in the isoforms expressed and in the presence of non-specific bands among the cell lines. We also analyzed the *MBNL1/Mbnl1* transcripts expressed in these cell lines by RT-PCR (Supplementary Figure S2). Within the same cell type, major isoform expression patterns were largely consistent between proteins and mRNA.

Typically, isoforms detected around 36 kDa are presumed to lack exon 3. While ab45899 can detect isoforms lacking exon 3, it showed no signal around 36 kDa in cell lines other than C2C12, suggesting that MBNL1_36_ and MBNL1_35_ are minimally expressed in these cells. In C2C12 cells, a transcript lacking exons 5, 7, and 8 was detected (Supplementary Figure S2, indicated by an asterisk), which may correspond to the strong ~35 kDa protein band observed in this cell line. However, when using 4A8 and 66837-1-Ig, which recognize exon 3, additional bands appeared around 36 kDa in THP-1, C2C12, and Neuro2a cells. The identity of these isoforms remains unknown. As noted earlier, ARP41227 recognizes isoforms containing exon 5, detecting bands around 42 kDa across all examined cell lines. Interestingly, additional bands were observed around ~25 kDa in the mouse-derived C2C12 and Neuro2a cells, though their identity remains unclear.

Since bands detected in the cell lines appeared not only near the expected molecular size of MBNL1 (approximately 40 kDa) but also at several other positions, there may be non-specific cross-reactivity. In particular, prominent bands were observed on the higher molecular weight side with ARP41227 and 66837-1-Ig. Therefore, knockdown experiments using siRNA targeting *MBNL1* were performed in HEK293 and Neuro2a cells (Figure 4B). A clear reduction in MBNL1 expression around 40 kDa was observed in both cell lines, whereas other bands did not exhibit a significant decrease. With ARP41227, strong bands were detected around 55 kDa or 70 kDa in both cell types, which were suggested to be non-specific. Similarly, a band at approximately 70 kDa detected by 66837-1-Ig in HEK293 cells was likely non-specific.

### 3.5. Detection of RNA Foci

**Figure 5:**
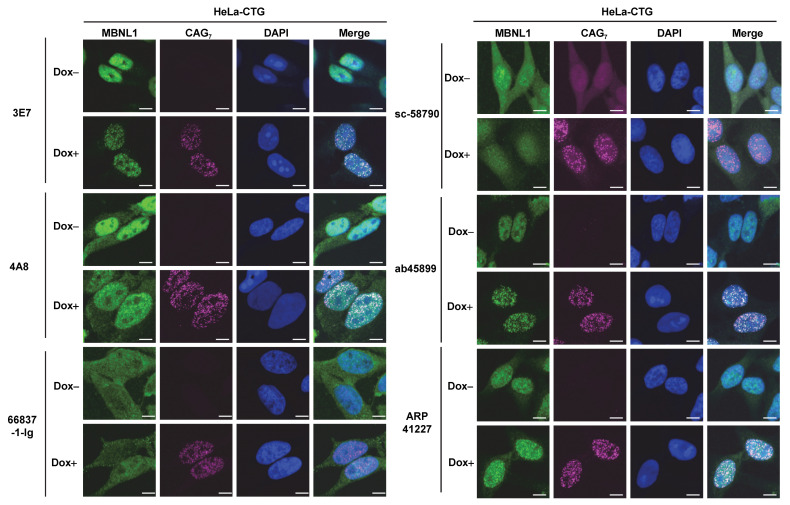
Detection of CUG repeat RNA foci in HeLa-CTG cells. CUG repeats were detected by RNA fluorescence in situ hybridization using the Cy5-2OMe-(CAG)7 probe. Immunofluorescences were performed using various MBNL1 antibodies to verify colocalization with CUG repeats. Scale bars: 10 μm.

RNA FISH and immunofluorescence staining for MBNL1 using six different antibodies were performed in HeLa-CTG cells, a DM1 model system. In HeLa-CTG cells, the addition of Dox induces the expression of the region spanning exon 14 to exon 15 of the *DMPK* gene, which contains expanded CUG repeats, leading to the formation of RNA foci. As a result, colocalization with RNA foci was confirmed for 3E7, 4A8, ab45899, and ARP41227 (Figure 5). In contrast, RNA foci colocalization was not observed with the other antibodies: 66837-1-Ig and sc-58790. We quantitatively analyzed colocalization using Pearson correlation coefficients between the fluorescence intensity of MBNL1 and RNA foci (Supplementary Figure S3), and 66837-1-Ig and sc-58790 showed significantly lower correlation coefficients than the other antibodies.

**Figure 6:**
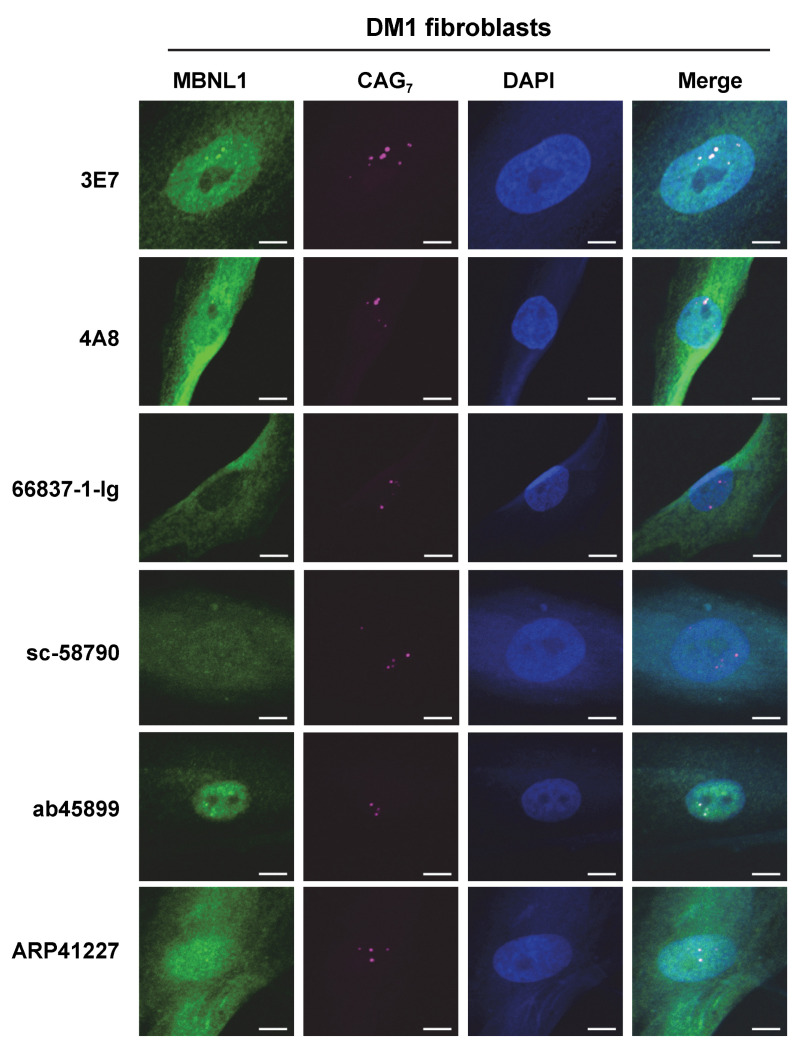
Fluorescence in situ hybridization analysis using DM1 fibroblasts. RNA foci were detected using the Cy5-2OMe-(CAG)7 probe. Immunofluorescences were performed using various MBNL1 antibodies to confirm colocalization with CUG repeat RNA foci. Scale bars: 10 μm.

Additionally, RNA FISH and immunofluorescence staining were conducted on fibroblasts derived from a DM1 patient (Figure 6), and it was found that 3E7 and ab45899 exhibited strong colocalization with RNA foci. While 4A8 and ARP41227 also showed partial colocalization, they displayed stronger cytoplasmic staining and higher nuclear background levels compared to 3E7 and ab45899. As observed in HeLa-CTG cells, 66837-1-Ig and sc-58790 did not show colocalization with RNA foci in DM1 patient fibroblasts, as also suggested by quantitative analysis (Supplementary Figure S3). The above analyses were conducted using paraformaldehyde-fixed cells. However, it has been reported that acetone–methanol fixation enhances the detection of MBNL1 localized to RNA foci by removing soluble MBNL1 [32,33]. We performed FISH-IF analysis on acetone–methanol-fixed HeLa-CTG cells and DM1 fibroblasts (Supplementary Figure S4). Compared to paraformaldehyde fixation, the detection of MBNL1 in RNA foci was particularly improved in DM1 fibroblasts. Even under these conditions, 66837-1-Ig and sc-58790 did not detect MBNL1 in RNA foci. Quantitative analysis also revealed significant differences among the tested antibodies (Supplementary Figure S4). These findings indicate that the ability of MBNL1 antibodies to detect RNA foci remains consistent across different cell types and fixation conditions, demonstrating a specific and reproducible recognition pattern.

### 3.6. Detection of MBNL1 by Immunoprecipitation

**Figure 7:**
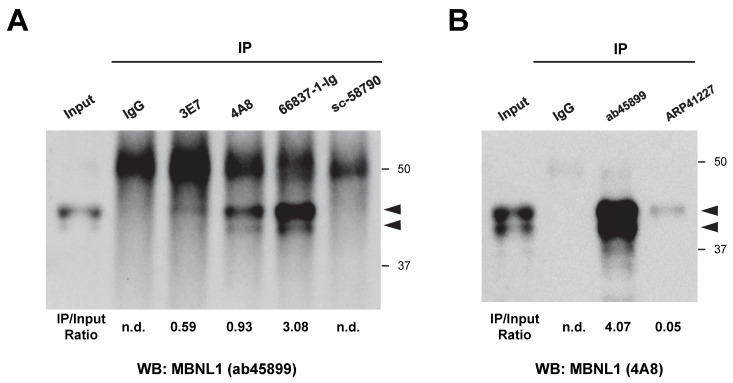
Immunoprecipitation experiments using various MBNL1 antibodies. (**A**) Immunoprecipitation using mouse-derived MBNL1 antibodies. MBNL1 was detected via Western blotting using ab45899. (**B**) Immunoprecipitation using rabbit-derived MBNL1 antibodies. MBNL1 was detected via Western blotting using 4A8. Arrowheads indicate the positions of MBNL1. The values shown below the image represent the band intensities obtained by immunoprecipitation, normalized to the band intensity of the input. n.d.: not detected.

The authors recently conducted immunoprecipitation of MBNL1 using the ab45899 antibody [31]. Here, we aimed to determine whether endogenous MBNL1 in HEK293 cells could be immunoprecipitated using various antibodies, followed by analysis via Western blotting. Experiments were conducted separately based on whether the primary antibody was derived from mice (Figure 7A) or rabbits (Figure 7B). The immunoprecipitated products were subsequently separated by SDS-PAGE, and Western blotting was used to detect MBNL1. As a result, MBNL1 bands were detected using five antibodies, except for sc-58790 (Figure 7A,B). Among them, however, ARP41227 yielded only a faint band. sc-58790 did not yield specific bands, indicating that it was unable to immunoprecipitate MBNL1 (Figure 7A).

## 4. Discussion

Accurate detection of endogenous MBNL1 using specific antibodies is essential for the development of therapeutic strategies that target toxic RNA or prevent MBNL1 sequestration into RNA foci. In this study, we compared and evaluated antibodies against MBNL1, aiming to identify their recognition sites, assess their ability to detect colocalization with RNA foci observed in DM1, and determine their suitability for immunoprecipitation. Six antibodies were tested, revealing that different antibodies recognize distinct regions of MBNL1. Since the detection of each MBNL1 isoform depends on the presence of its respective antibody recognition site, careful selection of antibodies is necessary.

Using deletion mutant analysis, we found that 3E7, 4A8, and 66837-1-Ig recognize amino acids 139–161 within exon 3, ab45899 targets amino acids 299–308 within exon 6, and ARP41227 binds to amino acids 270–275 within exon 5. Based on these results, we can predict MBNL1 isoforms that are detected by the above antibodies (Supplementary Figure S5). We also examined the reactivity of these antibodies against MBNL2 and MBNL3, finding that only sc-58790, originally marketed as an MBNL1/2/3 antibody, reacted with these paralogs. However, sc-58790 exhibited the strongest affinity for MBNL2.

The suitability of detecting the colocalization of MBNL1 in RNA foci varied depending on the antibody used. Consistent results were obtained in both HeLa-CTG cells and fibroblasts derived from DM1 patients, though some antibodies showed reduced recognition in patient-derived cells. No clear correlation was observed between recognition sites and RNA foci detection ability, indicating that the selection of antibodies for foci detection should be based on functional evaluation.

Certain antibodies (3E7, 4A8, 66837-1-Ig, and ab45899) were found to be suitable for immunoprecipitation, whereas ARP41227 and sc-58790 were deemed unsuitable for this application. These antibodies showed non-specific cross-reactivity (ARP41227) or higher affinity for MBNL2 (sc-58790), which may underlie their weaker interaction with MBNL1. Similar to RNA foci detection, there was no evident correlation between recognition sites and the ability to perform immunoprecipitation.

The results are summarized in Table 2. Among the tested antibodies, ab45899 demonstrated broad isoform recognition and was suitable for both RNA foci detection and immunoprecipitation, making it the most versatile option. However, certain isoforms, such as the 40s variant, may not be detected by ab45899. For example, in THP-1 cells, some bands detected by 4A8 and 66837-1-Ig were not detected by ab45899, highlighting the need for further investigation to determine their identity. In addition, 3E7 and 4A8 also demonstrated minimal non-specific bands in Western blotting and were effective for RNA foci detection and immunoprecipitation, making them reliable antibodies. However, since these antibodies recognize exon 3, they are not suitable for detecting isoforms lacking this exon. This study also identified antibodies that specifically recognize unique regions of MBNL1. One such antibody is ARP41227, which was shown to target the N-terminal region of exon 5 involved in nuclear localization. Notably, ARP41227 did not react with the corresponding region of MBNL2. Although this antibody successfully detected the colocalization of endogenous MBNL1 with RNA foci in RNA overexpression systems, it displayed significant non-specific bands in the Western blot analysis. Exon 5 inclusion is suppressed by MBNL1 autoregulation but increases in DM1 and DM2 due to MBNL1 sequestration into RNA foci, which impairs its function. Given this, ARP41227 may serve as a potential tool for detecting such pathological changes.

In this study, we analyzed the expression profiles of MBNL1 isoforms in several cultured cell lines using Western blotting with various MBNL1 antibodies. Although not all detected isoforms were fully identified, the combined use of RT-PCR and sequencing analysis is expected to contribute to a more precise understanding of their expression patterns. Currently, more than 40 MBNL1 isoforms are registered in the NCBI gene database. This study has elucidated the exons recognized by each antibody, which will aid in distinguishing the isoforms expressed in different cell types. Many MBNL1 isoforms commonly contain exon 2 or exon 4. Regarding exon 4, sc-58790 was found to recognize it; however, its specificity for MBNL1 is low. In the future, the development of antibodies targeting these exons is anticipated to facilitate more comprehensive detection of MBNL1.

In summary, this study provided a comparative analysis of commercially available MBNL1 antibodies and elucidated their characteristics. These findings provide a valuable foundation for research and interpretation related to MBNL1-associated diseases, including myotonic dystrophy, as well as studies on RNA metabolism involving MBNL1.

## 5. Conclusions

This study provides a comparative analysis of antibodies, elucidating their recognition characteristics and suitability for different applications. We also emphasize the diversity of MBNL1 isoforms, which display cell-line-specific expression patterns and distinct antibody reactivity. These findings provide a valuable foundation for selecting the appropriate antibodies for research on diseases associated with MBNL1 and RNA metabolism.

## Figures and Tables

**Table 1:** Antibodies used in this study.

MBNL1 Antibody	Company	Catalog Number	Host Species	Immunogen
3E7	Abnova (Taipei, Taiwan)	H00004154-M02	Mouse monoclonal	Full-length recombinant human MBNL1
4A8	Santa Cruz Biotechnology (Dallas, TX, USA)	sc-136165	Mouse monoclonal	Full-length recombinant human MBNL1
MBNL1/2/3	Santa Cruz Biotechnology	sc-58790	Mouse monoclonal	A 371 amino acid fragment of human MBNL1
66837-1-Ig	Proteintech (Rosemont, IL, USA)	66837-1-Ig	Mouse monoclonal	Recombinant protein (amino acids 59–400 of human MBNL1_43_)
ab45899	Abcam (Cambridge, UK)	ab45899	Rabbit polyclonal	Synthetic peptide (amino acids 250–350 of human MBNL1)
ARP41227	Aviva Systems Biology (San Diego, CA, USA)	ARP41227_P050	Rabbit polyclonal	Synthetic peptide (amino acids 268–317)

**Table 2:** Summary of findings in this study.

MBNL1 Antibody	Recognition Site	RNA Foci Detection	Immunoprecipitation
3E7	139–161 (exon 3)	Applicable	Applicable
4A8	139–161 (exon 3)	Applicable	Applicable
sc-58790	184–238 (exon 4)	Not applicable	Not applicable
66837-1-Ig	139–161 (exon 3)	Not applicable	Applicable
ab45899	299–308 (exon 6)	Applicable	Applicable (recommended)
ARP41227	270–275 (exon 5)	Applicable	Applicable (not efficient)

## Data Availability

All data supporting the findings of this study are available from the corresponding authors upon reasonable request.

## Supplementary Materials

The following supporting information can be downloaded at https://www.mdpi.com/article/10.3390/genes16060658/s1, Figure S1: Identification of ARP41227 recognition sites using MBNL1 deletion mutants; Figure S2: Isoforms of MBNL1/Mbnl1 expressed in the cell lines used in this study; Figure S3: Quantitative analysis of MBNL1-RNA foci colocalization; Figure S4: FISH-IF analysis of cells treated with acetone–methanol fixation; Figure S5: Predicted reactivity of various MBNL1 antibodies toward MBNL1 isoforms; Figure S6: Uncropped original images.

## Abbreviations

The following abbreviations are used in this manuscript:

DM	Myotonic dystrophy
DM1	Myotonic dystrophy type 1
DM2	Myotonic dystrophy type 2
EGFP	Enhanced green fluorescent protein
FISH	Fluorescence In Situ Hybridization
IF	Immunofluorescence
MBNL1	Muscleblind-like 1
ZnF	Zinc finger
