# Supplementary material for: Comparative Analysis of MBNL1 Antibodies: Characterization of Recognition Sites and Detection of RNA Foci Colocalization
Source: Genes (Basel). 2025 May 29;16(6):658. doi: 10.3390/genes16060658 (PMC12192522; doi:10.3390/genes16060658)
Supplement: Supplementary file 1 [file genes-16-00658-s001.zip › genes-3652691-supplementary.pdf]

Figure S1

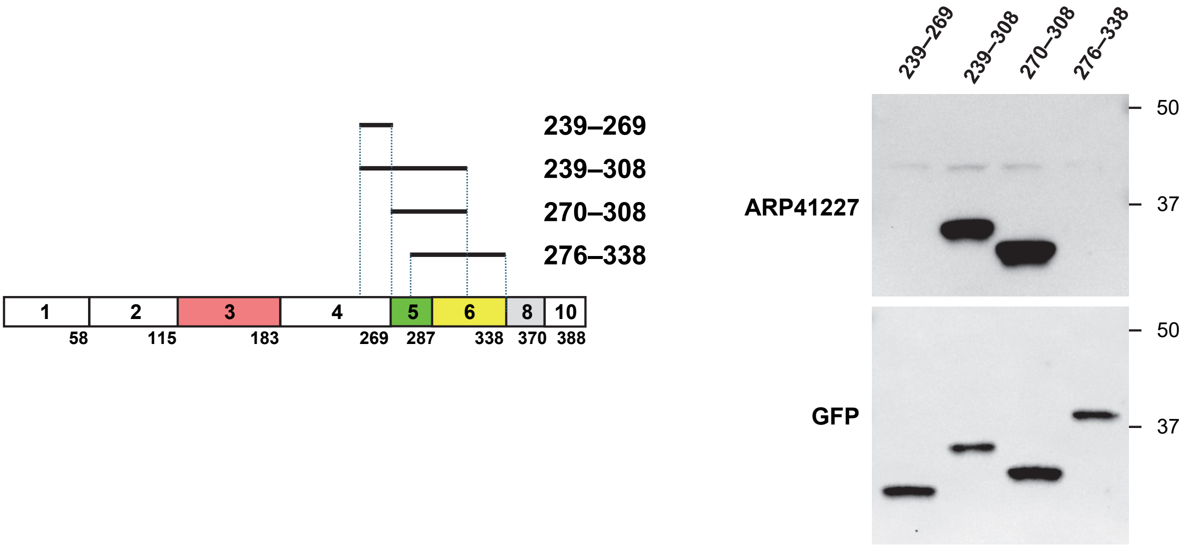

Figure S1. Identification of ARP41227 recognition sites using MBNL1 deletion mutants
The reactivity of ARP41227 toward the deletion mutants illustrated in the schematic diagram was analyzed by Western blotting, following the same approach as in Figure 2.

Figure S2

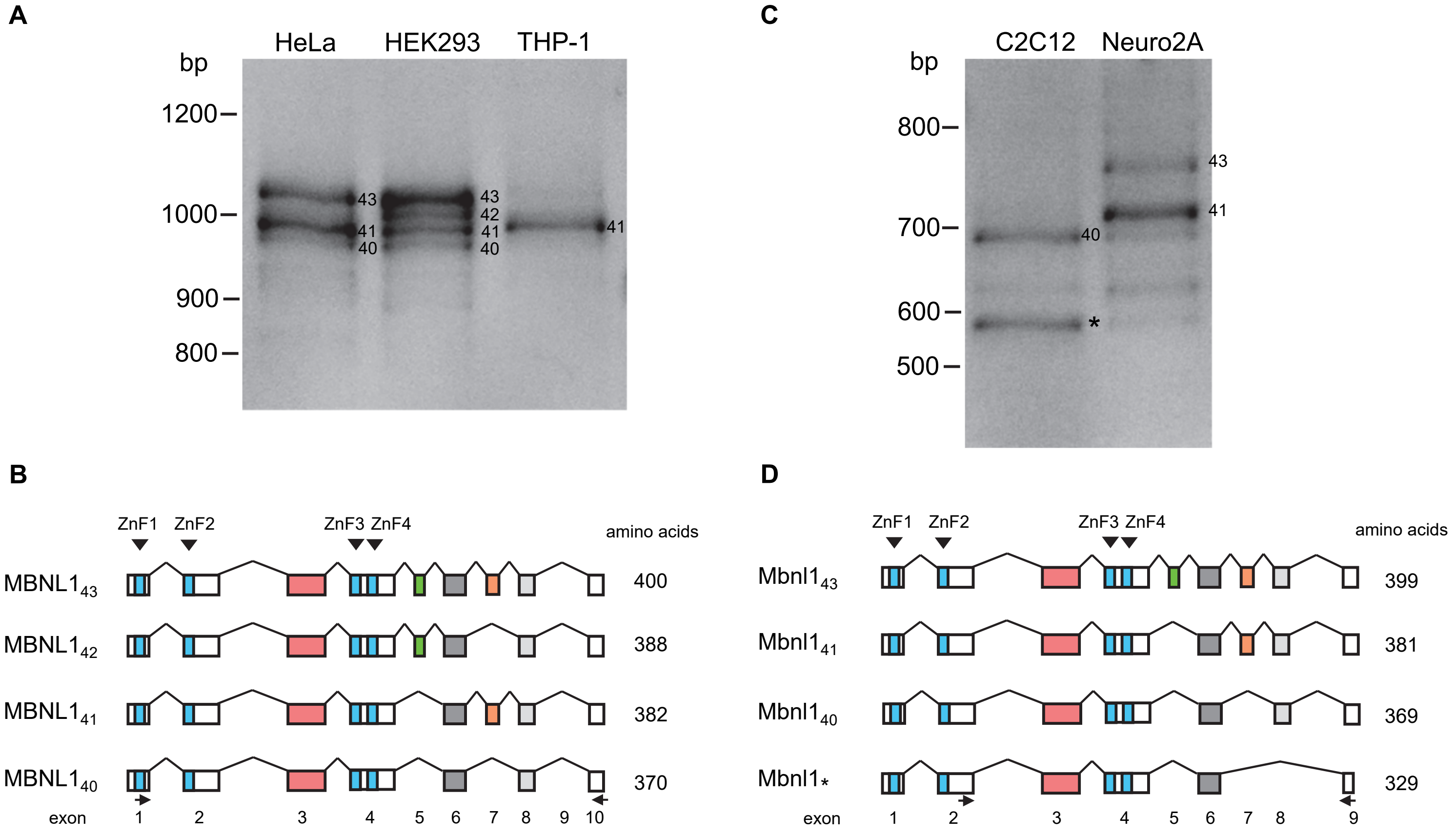

Figure S2. Isoforms of MBNL1/Mbnl1 expressed in the cell lines used in this study.
Reverse transcription-PCR products were separated by 8% polyacrylamide gel electrophoresis.
A, **B**. Schematic representation of MBNL1 transcripts and their corresponding isoforms expressed in human-derived cell lines. Black arrows indicate the forward and reverse primer sites.
C, **D**. Schematic representation of Mbnl1 transcripts and their corresponding isoforms expressed in mouse-derived cell lines. Black arrows indicate the forward/reverse primer sites. The asterisk in C2C12 indicates an isoform lacking exons 5, 7, and 8. Exon 9 is shorter in this isoform than in other isoforms due to a frameshift.

Figure S3

A

HeLa-CTG cells (PFA fixation)

Pearson correlation coefficients

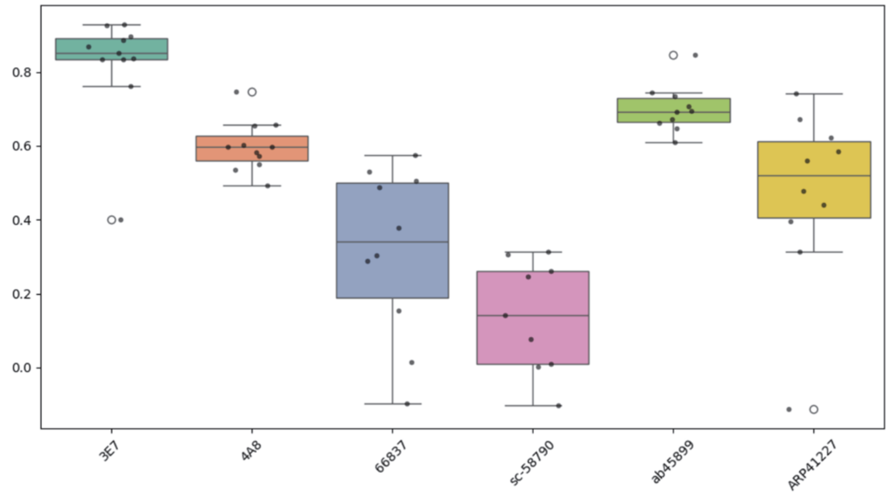

P-values for pairwise comparisons

	3E7	4A8	66837	ARP41227	ab45899
sc-58790	<0.00001	0.02816	1.00000	0.45797	0.00017
ab45899	1.00000	1.00000	0.00547	0.32826	
ARP41227	0.00940	1.00000	1.00000		
66837	0.00004	0.39061			
4A8	0.15792				

B

DM1 fibroblasts (PFA fixation)

Pearson correlation coefficients

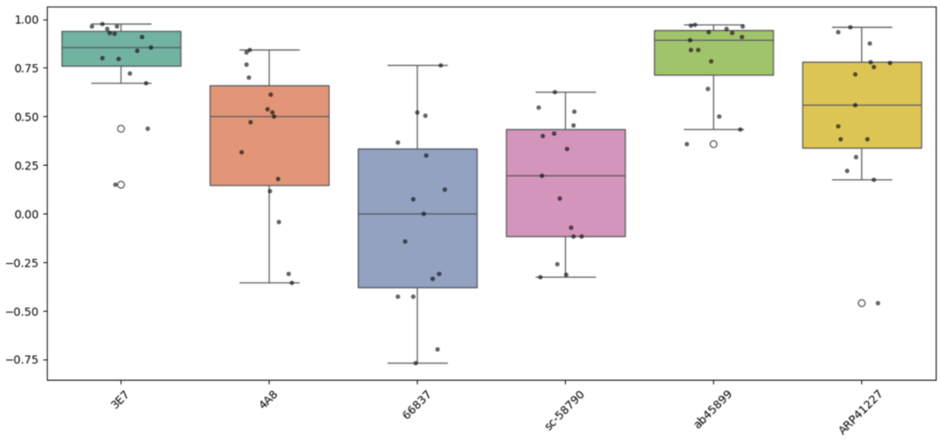

P-values for pairwise comparisons

	3E7	4A8	66837	ARP41227	ab45899
sc-58790	0.00033	1.00000	1.00000	0.50440	0.00032
ab45899	1.00000	0.04364	0.00001	0.50440	
ARP41227	0.51322	1.00000	0.06823		
66837	0.00001	0.70758			
4A8	0.04465				

Figure S3. Quantitative analysis of MBNL1-RNA foci colocalization.
Pearson correlation coefficients and pairwise comparisons of MBNL1 antibodies in paraformaldehyde-fixed HeLa-CTG cells (**A**) and DM1-derived fibroblasts (**B**) are shown.

Figure S4

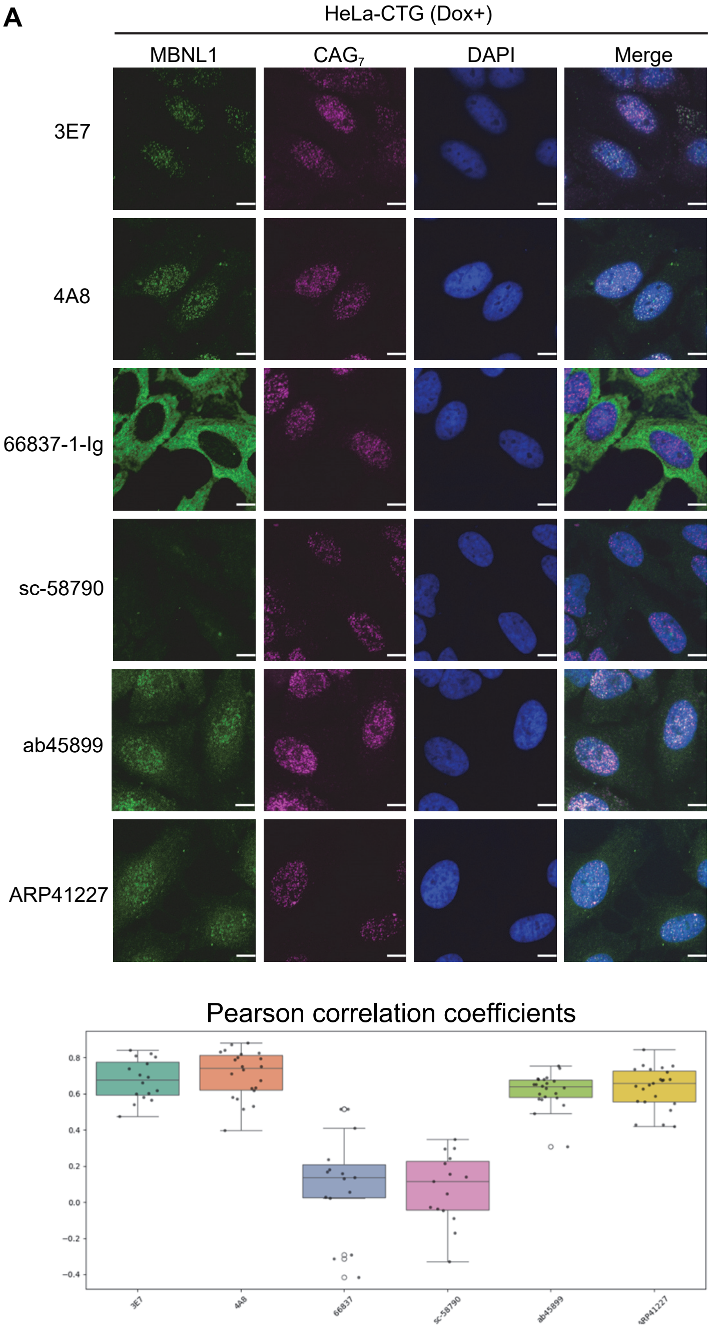

P-values for pairwise comparisons

	3E7	4A8	66837	ARP41227	ab45899
sc-58790	<0.00001	<0.00001	1.00000	0.00002	0.00012
ab45899	1.00000	1.00000	0.00034	1.00000	
ARP41227	1.00000	1.00000	0.00005		
66837	0.00002	<0.00001			
4A8	1.00000				

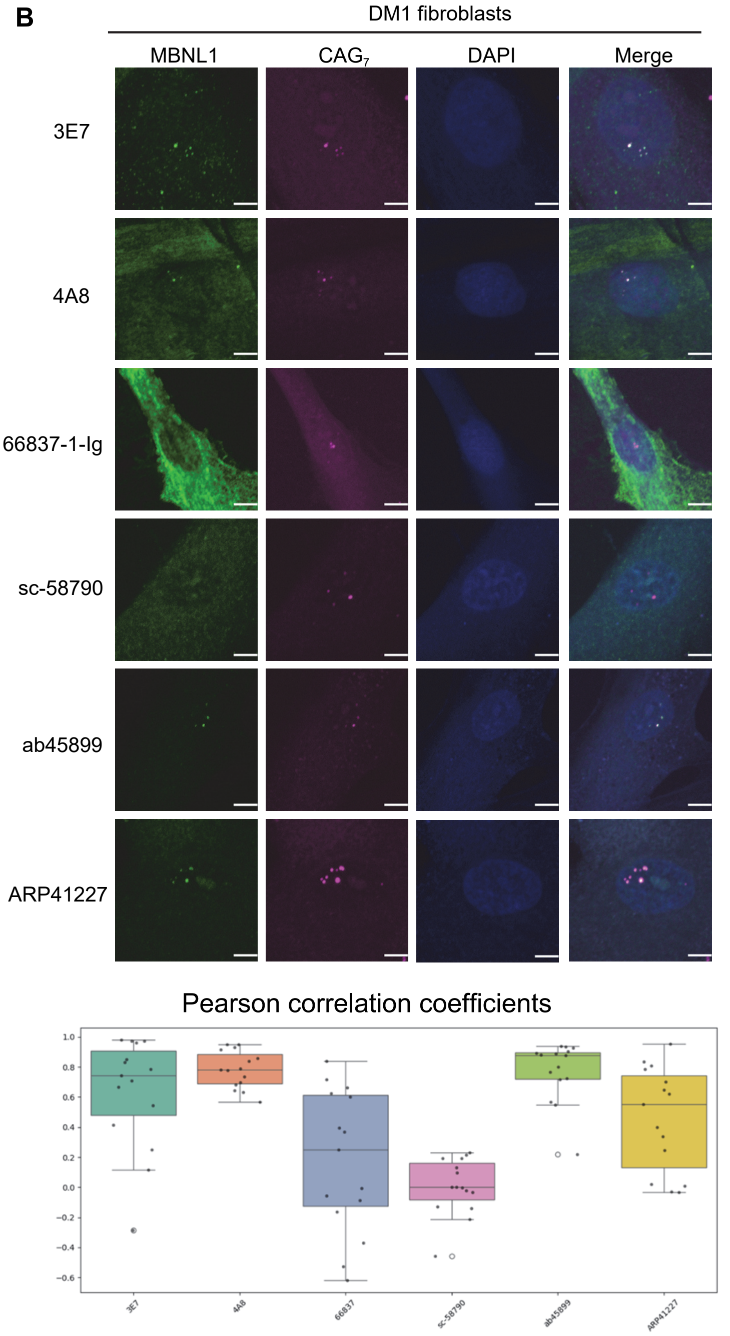

P-values for pairwise comparisons

	3E7	4A8	66837	ARP41227	ab45899
sc-58790	0.00048	0.00002	1.00000	0.12375	0.00001
ab45899	1.00000	1.00000	0.00145	0.29379	
ARP41227	1.00000	0.39388	1.00000		
66837	0.03085	0.00228			
4A8	1.00000				

Figure S4. FISH-IF analysis of cells treated with acetone-methanol fixation.
FISH-IF images, Pearson correlation coefficients, and pairwise comparisons of MBNL1 antibodies in acetone-methanol-fixed HeLa-CTG cells (**A**) and DM1-derived fibroblasts (**B**) are shown. Scale bars: 10 μm.

Figure S5

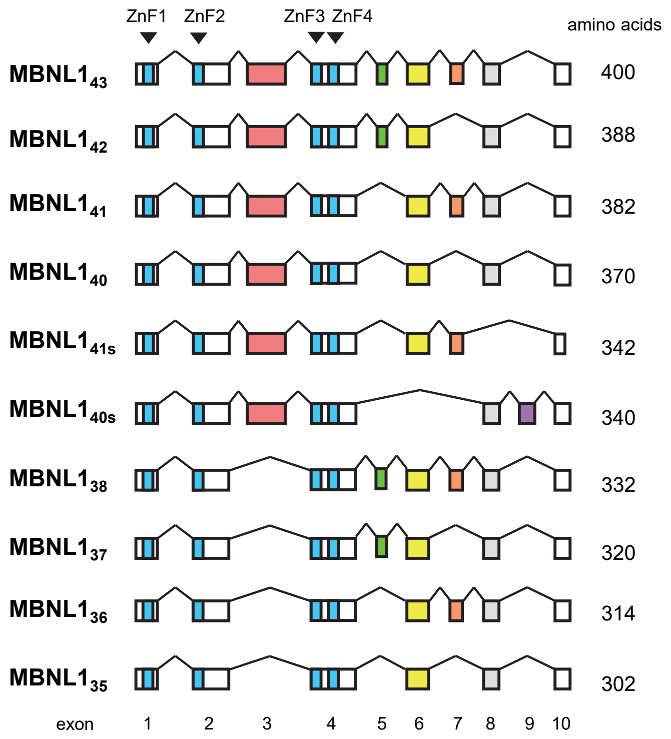

Predicted antibody reactivity in Western blot analysis

	3E7	4A8	66837-1-Ig	sc-58790	ab45899	ARP41227
MBNL1$_{43}$	✔	✔	✔	✔	✔	✔
MBNL1$_{42}$	✔	✔	✔	✔	✔	✔
MBNL1$_{41}$	✔	✔	✔	✔	✔	
MBNL1$_{40}$	✔	✔	✔	✔	✔	
MBNL1$_{41s}$	✔	✔	✔	✔	✔	
MBNL1$_{40s}$	✔	✔	✔	✔		
MBNL1$_{38}$				✔	✔	✔
MBNL1$_{37}$				✔	✔	✔
MBNL1$_{36}$				✔	✔	
MBNL1$_{35}$				✔	✔	

Figure S5. Predicted reactivity of various MBNL1 antibodies toward MBNL1 isoforms.
Based on the results of this study, MBNL1 antibodies predicted to detect specific MBNL1 isoforms are indicated with check marks.

Figure S6

Fig.1B.

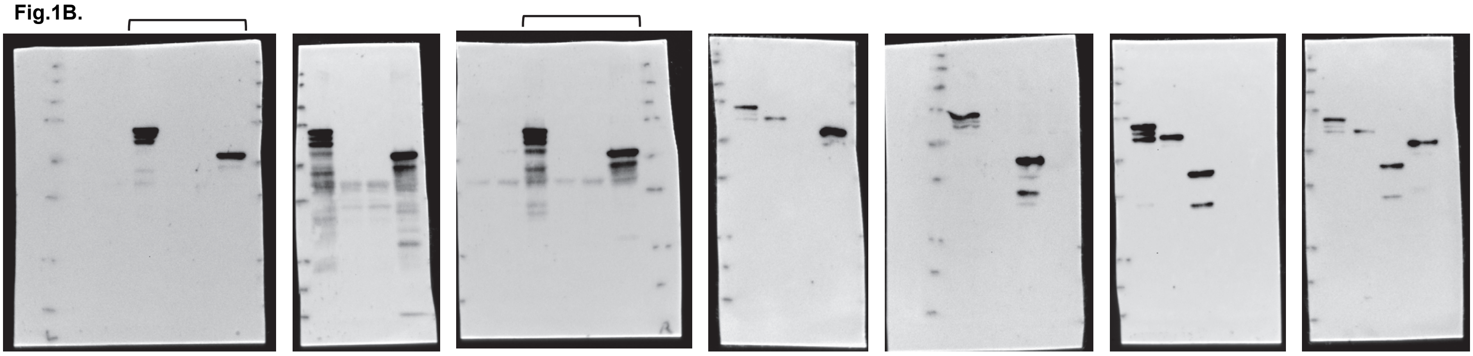

3E7 | 4A8 | 66387-1-Ig | sc-58790 | ab45899 | ARP41227 | GFP

Fig.2A.

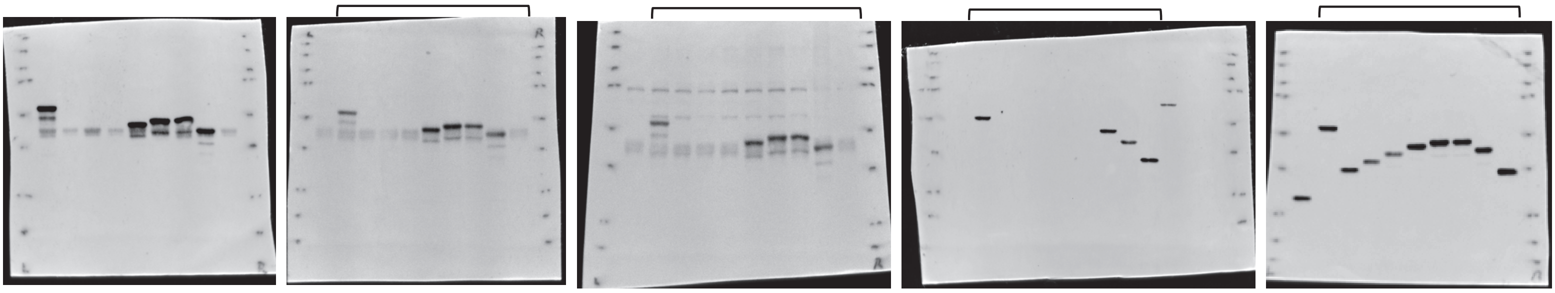

3E7 | 4A8 | 66387-1-Ig | sc-58790 | GFP

Fig.2B.

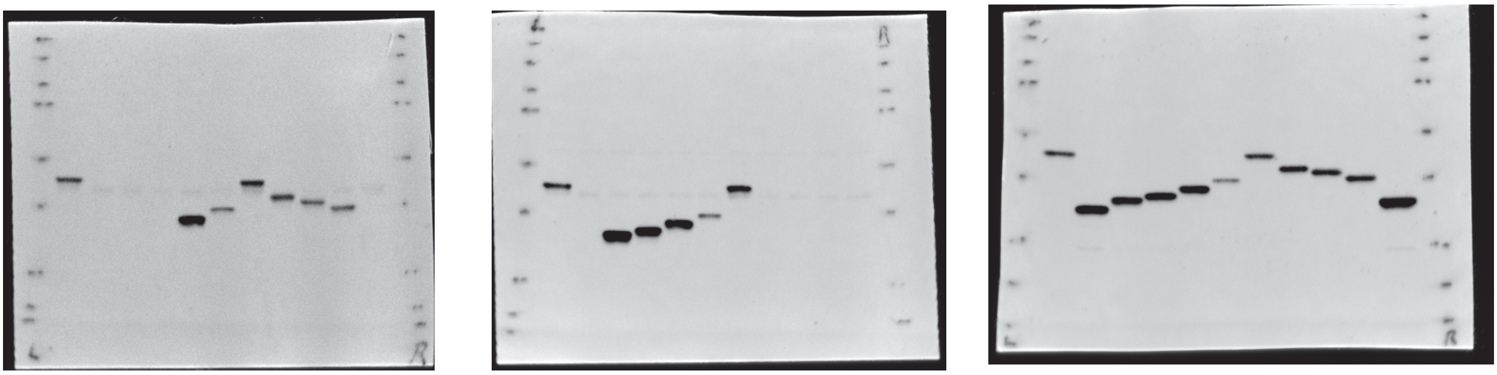

ab45899 | ARP41227 | GFP

Fig.3B.

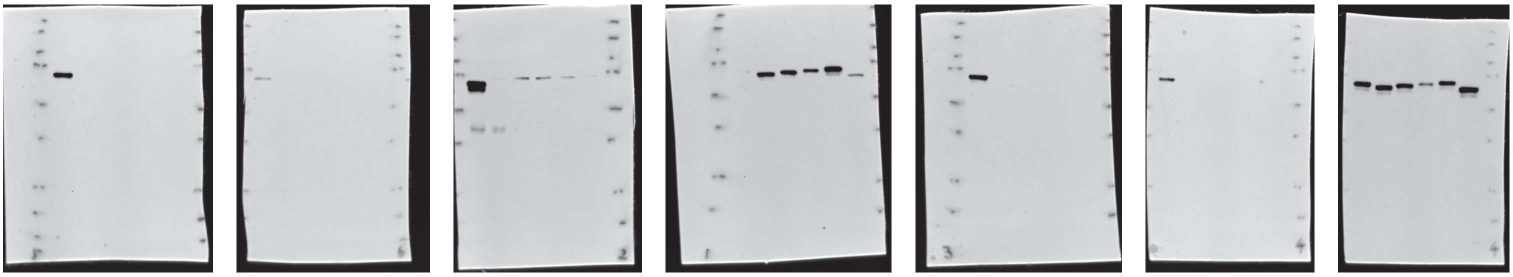

3E7 | 4A8 | 66387-1-Ig | sc-58790 | ab45899 | ARP41227 | GFP

Figure S6 (continued)

Fig.4A.

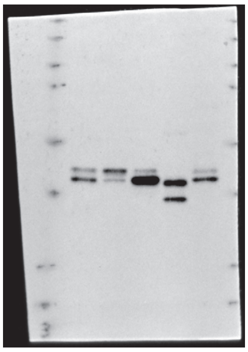

3E7

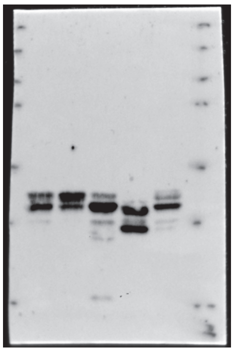

4A8

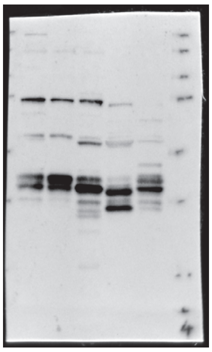

66387-1-Ig

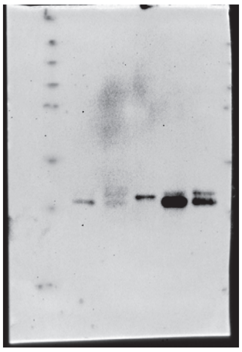

sc-58790

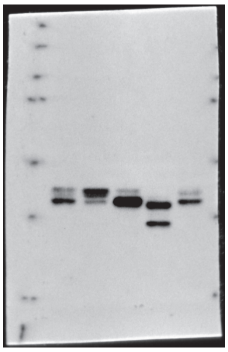

ab45899

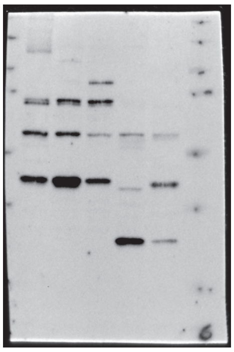

ARP41227

Fig.4B.

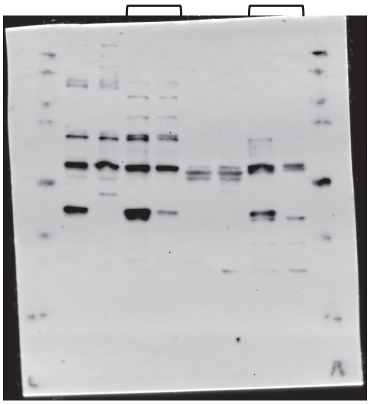

ARP41227

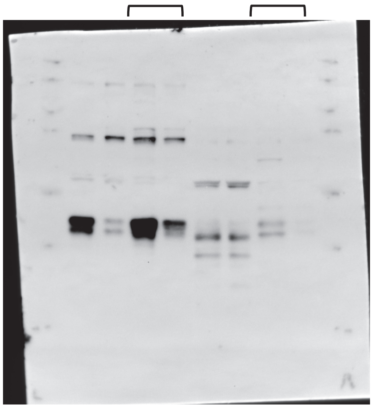

66387-1-Ig

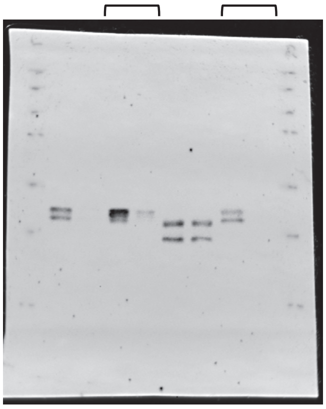

4A8

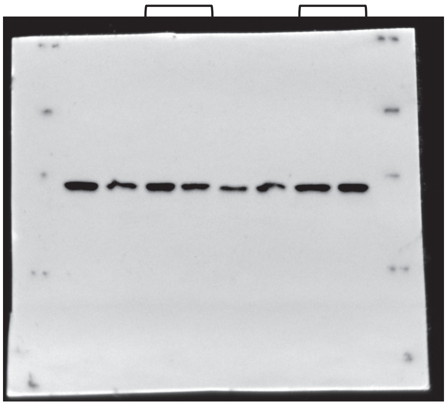

GAPDH

Fig.7A.

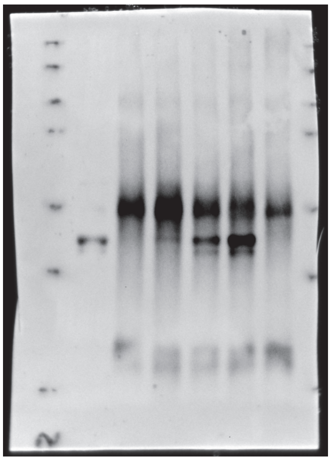

ab45899

Fig.7B.

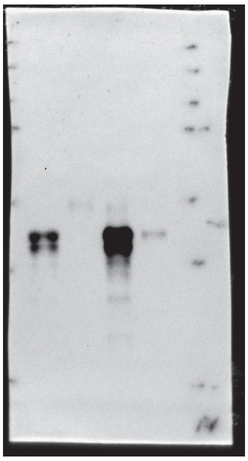

4A8

Fig.S1A.

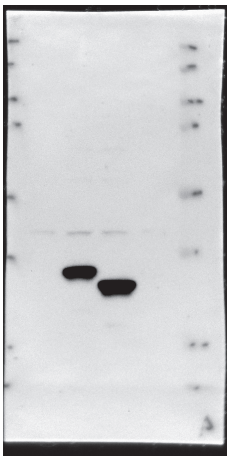

ARP41227

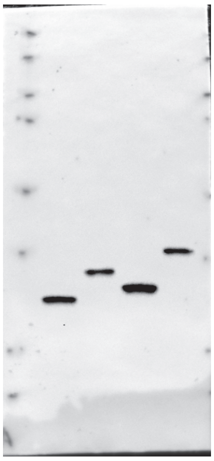

GFP

Fig.S2A. **Fig.S2C.**

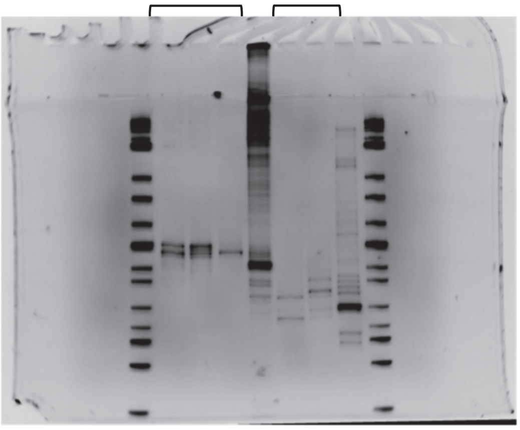

Figure S6. Uncropped Original Images.
